# A series of decision-theoretic phase II trials of related treatments

**DOI:** 10.1186/1745-6215-14-S1-O8

**Published:** 2013-11-29

**Authors:** Siew Wan Hee, Nicholas Parsons, Matthew Costa, Nigel Stallard

**Affiliations:** 1 University of Warwick, Coventry, UK

## 

It is not uncommon for a pharmaceutical company to develop several new drugs simultaneously for the same indication. In a small population it may be infeasible to try all new drugs concurrently. A practical approach is to consider one at a time and if the drug is recommended for a phase III trial to temporarily suspend development of other drugs.

We propose a development plan that encompasses phase II and III trials, considering a series of sequential phase II trials with interim decision-making. At each stage of a phase II trial, a decision is made whether to continue with participant recruitment to the current trial, stop the current trial and recommend the drug to be tried in a phase III trial, stop the current trial and initiate a new phase II trial with a different drug or stop the current trial and abandon the development.

The unknown treatment effect for each drug is assumed to follow a prior distribution. In addition, as drugs are intended for the same population it is not unrealistic to consider treatment effects to be correlated, so that the prior distribution will reflect this. As data are observed from preceding and current trials, prior densities for subsequent drugs are updated, informing us of the performance of the groups of drugs under evaluation. The application of the design is illustrated in the setting of severe arthritis of the hip trials.

